# The Effects of Various Conditions of Short-Term Rejuvenation Heat Treatment on Room-Temperature Mechanical Properties of Thermally Aged P92 Boiler Steel

**DOI:** 10.3390/ma14206076

**Published:** 2021-10-14

**Authors:** Ladislav Falat, Lucia Čiripová, Viera Homolová, Miroslav Džupon, Róbert Džunda, Karol Kovaľ

**Affiliations:** Institute of Materials Research, Slovak Academy of Sciences, Watsonova 47, 04001 Košice, Slovakia; lciripova@saske.sk (L.Č.); vhomolova@saske.sk (V.H.); mdzupon@saske.sk (M.D.); rdzunda@saske.sk (R.D.); kkoval@saske.sk (K.K.)

**Keywords:** thermal embrittlement, Laves phase, rejuvenation heat treatment, impact toughness, static tensile properties

## Abstract

In this work, the effects of various conditions of short-term rejuvenation heat treatment on room-temperature mechanical properties of long-term aged P92 boiler steel were investigated. Normalized and tempered P92 steel pipe was thermally exposed at 600 °C for time durations up to 5000 h in order to simulate high-temperature material degradation, as also occurring in service conditions. Thus, thermally embrittled material states of P92 steel were prepared, showing tempered martensitic microstructures with coarsened secondary phase precipitates of Cr_23_C_6_-based carbides and Fe_2_W-based Laves phase. Compared with the initial normalized and tempered material condition, thermally aged materials exhibited a slight decrease in strength properties (i.e., yield stress and ultimate tensile strength) and deformation properties (i.e., total elongation and reduction of area). The hardness values were almost unaffected, whereas the impact toughness values showed a steep decrease after long-term ageing. An idea for designing the rejuvenation heat treatments for restoration of impact toughness was based on tuning the material properties by short-term annealing effects at various selected temperatures somewhat above the long-term ageing temperature of P92 material. Specifically, the proposed heat treatments were performed within the temperature range between 680 °C and 740 °C, employing variable heating up and cooling down conditions. It was revealed that short-term annealing at 740 °C for 1 h with subsequent rapid cooling into water represents the most efficient rejuvenation heat treatment procedure of thermally aged P92 steel for full restoration of impact toughness up to original values of normalized and tempered material state. Microstructural observations clearly indicated partial dissolution of the Laves phase precipitates to be the crucial phenomenon that played a key role in restoring the impact toughness.

## 1. Introduction

P/T92 grade (EN X10CrWMoVNb9-2) creep-resistant steel belongs to a group of modern, high-alloyed structural materials, typically used in high-efficiency power engineering [1,2,3]. It is frequently utilized for constructing the high-temperature boiler components of supercritical and ultra-supercritical power plants. Its initial normalized and tempered (N&T) microstructure, corresponding to industrially produced material condition, typically consists of tempered martensite, i.e., ferrite with secondary phase precipitates of intergranular Cr_23_C_6_-based carbides and intragranular (V,Nb)(C,N) type carbonitrides [4,5,6]. Thanks to a high amount of chromium (around 9 wt.% Cr) in P/T92 material, the Cr_23_C_6_-based carbides represent major grain-boundary precipitates, enhancing the steel creep strength. Tungsten is another important alloying element, which highly exceeds its solubility limit in the ferrite solid solution of the P/T92 steel matrix. As a consequence, gradual precipitation of intermetallic Fe_2_W-based Laves phase occurs in P/T92 steel during its long-term high-temperature exposure [7,8,9,10,11,12]. In numerous published works [7,8,9,10,11,12,13,14], the degradation of mechanical properties (especially creep strength and impact toughness) of P/T92 steel was primarily ascribed to the Laves phase precipitation and coarsening. Detailed microstructural analyses, e.g., in [10,11,12,13,14,15,16], confirmed that the Laves phase very often precipitates on the Cr_23_C_6_-based carbides, which serve as preferential sites for the Laves phase nucleation. Some authors, e.g., [17,18,19,20], revealed positive effects of fine Laves phase particles on creep resistance of high-chromium boiler steels in the early stages of their high-temperature exposure. However, with the increasing exposure time, the localities of thermally coarsened Laves phase gradually become the creep cavities’ nucleation sites [5,13,15]. In earlier experimental works [21,22], the research was focused on the effect of Laves phase on impact brittleness. These works reported a notable decrease in the impact toughness values of thermally exposed materials after the Laves phase precipitation. Another study [23] focused on the possibilities of improvement or even restoration of original values of impact toughness by employing short-term and long-term heat treatments of thermally exposed material states at temperatures above the Laves phase precipitation. It has been shown that, by dissolution of the Laves phase, it is really possible to achieve impact toughness values at the level of the original material state. The works of [23,24] dealt with the effects of the morphology, size, and volume fraction of Laves phase precipitates on the notch sensitivity and quite satisfactory correlation has been found between impact brittleness of studied materials and the volume fraction of the intermetallic phase. All the aforementioned findings seem to support the generally accepted opinion on the Laves phase to be considered the main embrittling factor in high-chromium boiler steels. However, the more reserved opinions regarding the Laves phase consider its rather supplementary effect on the processes of creep-resistant steels’ degradation [25,26]. Especially, a recent study [26] focused on the evaluation of the Laves phase effect on impact brittleness of P92 steel gave rise to an even more open approach regarding this issue. In this study, it has been reported that, by short-term heat treatment (700 °C/1 h) of the thermally exposed state (600 °C/2035 h) of P92 steel with the presence of the Laves phase in microstructure, it was possible to suppress thermal embrittlement of the material and restore its original impact toughness. Moreover, it was shown that the described improvement of impact toughness could be obtained despite negligible changes in the Laves phase precipitation state. Based on the achieved results, the authors of this study [26] emphasized the need for continuation of more detailed research about the origin of impact brittleness of thermally exposed 9Cr creep-resistant steels.

Our current research is aimed at the investigation of the effects of various conditions of short-term rejuvenation heat treatment on the room-temperature mechanical properties of thermally aged P92 boiler steel and seeking feasible ways for its impact toughness restoration while maintaining the strength and hardness.

## 2. Experimental Materials and Methods

The experimental material used in the present study was the P92 grade (EN X10CrWMoVNb9-2) boiler steel in the form of a seamless pipe with an outer diameter of 76 mm and a wall thickness of 12 mm. Its chemical composition is listed in Table 1.

**Figure 1:**
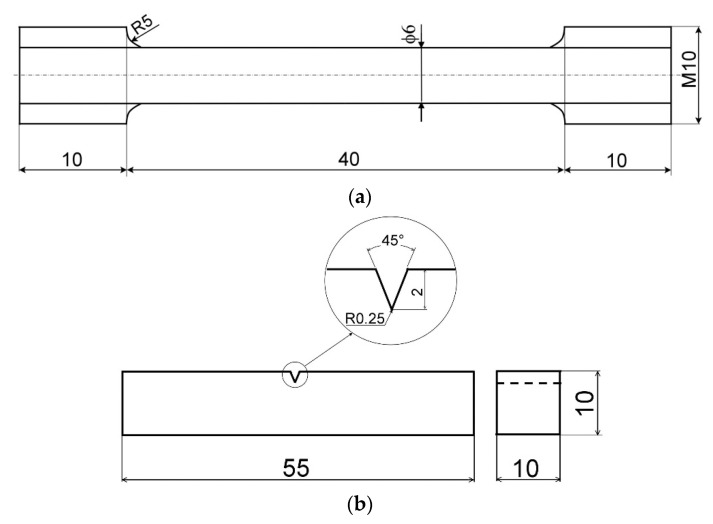
Schemes of test specimens used for individual mechanical tests: (**a**) uniaxial tensile test specimen and (**b**) Charpy V-notch impact bending test specimen. All dimensions are in mm.

The experimental material was received in conventional normalized and tempered (N&T) material condition, i.e., 1060 °C/1 h/air, 760 °C/2 h/air. One portion of the material (pipe segment) was studied in this initial N&T material state. The other two portions of the material were individually subjected to long-term ageing at 600 °C for 2500 h (600 °C/2.5 kh) and 5000 h (600 °C/5 kh). The ageing treatments were performed within an electric resistance furnace LAC PKE 18/12R (LAC, s.r.o., Rajhrad, Czech Republic). The individual material states (N&T, 600 °C/2.5 kh, and 600 °C/5 kh) were characterized by means of metallographic analyses using scanning electron microscope (SEM) JEOL JSM-7000F (Jeol Ltd., Tokyo, Japan), hardness measurements using Vickers hardness tester 432 SVD (Wolpert Wilson Instruments, division of Instron Deutschland GmbH, Aachen, Germany), tensile tests using universal testing machine TIRATEST 2300 (TIRA GmbH, Schalkau, Germany), and Charpy V-notch (CVN) impact bending tests using conventional Charpy pendulum impact tester PSW 30 (VEB Werstoffprüfmaschinen Leipzig, Leipzig, Germany) with a 300 J impact energy pendulum hammer. The hardness measurements were performed on plain surfaces of conventionally prepared metallographic cross sections at 9.8 N loading for 10 s per measurement. The cross-head speed by quasi-static tensile testing and impact speed by dynamic impact testing were 0.05 mm/min and 5.6 m/s, respectively. The used test specimens are schematically depicted in Figure 1. The tensile tests and tensile test specimens conformed to the standard ISO 6892-1:2019 [27]. The impact bending tests and impact test specimens conformed to the standard ISO 148-1:2016 [28].

**Figure 2:**
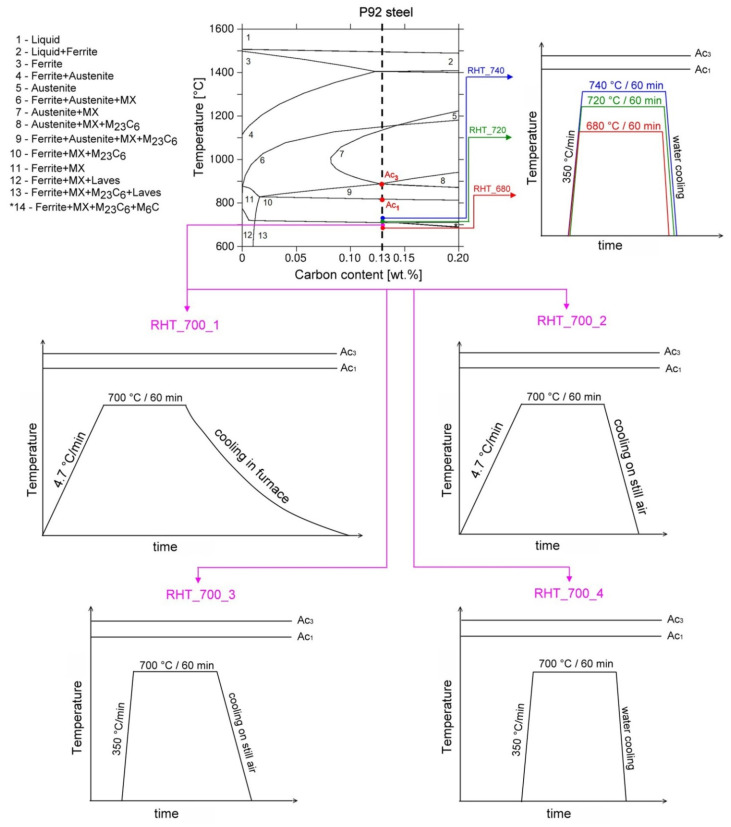
Experimental design philosophy coupling calculated equilibrium phase diagram with schematic illustrations of proposed short-term rejuvenation heat treatments of thermally aged P92 steel. The P92 steel composition is indicated in the diagram by the vertical dashed line at 0.13 wt.% C.

Next, the thermally embrittled material state, i.e., 600 °C/5 kh was selected for our further experiments, aimed at restoration of the P92 material impact toughness. Our experimental approach was based on tuning the precipitation state of major secondary phase precipitates in thermally embrittled material by additional short-term heat treatments. The corresponding annealing temperatures were selected somewhat above the long-term ageing temperature of P92 steel, but well below its ferrite-to-austenite transformation temperature (Ac_1_) to avoid the material over-tempering. Figure 2 shows schematic illustrations of individual proposed heat treatments in context with an equilibrium phase diagram including isoplethal section for P92 steel. The diagram was calculated using thermodynamic software Thermo-Calc (version S, Thermo-Calc Software AB, Solna, Sweden) employing non-commercial thermodynamic database STEEL16. The annealing temperatures of considered rejuvenation heat treatments were selected to be in range from 680 °C to 740 °C, lying in the vicinity of estimated solvus temperature of Laves phase (i.e., 704 °C) in present P92 material (see Figure 2).

At first, to follow the information from the work of Zhong et al. [26], the reheating at 700 °C for 1 h of thermally aged P92 steel was examined. No details about the heating up and cooling down conditions were given in [26]. Therefore, in our present study, four various heat treatments at 700 °C have been individually performed in order to examine various heating up and cooling down conditions of the rejuvenation procedure, aimed at anticipated improvement of impact toughness. Then, further heat treatments with most efficient heating up and cooling down conditions were individually performed at other three temperatures (i.e., 680 °C, 720 °C, and 740 °C) in order to search for the maximal rejuvenation effect. The holding time at the selected annealing temperature for each rejuvenation heat treatment (RHT) was always 1 h. More details about individual rejuvenation heat treatments are shown in Table 2.

All the heat treatments were performed within the electric resistance furnace, as specified above. Each individual RHT of thermally aged (600 °C/5 kh) P92 material was performed on six prismatic samples with dimensions of 11 × 11 × 60 mm. After performing the specific heat treatment, three conventional Charpy V-notch impact test specimens and three conventional cylindrical tensile test specimens (as shown in Figure 1) were prepared and subjected to corresponding mechanical tests as specified above. Supplementary microstructural and fractographic analyses were performed using the SEMs JEOL JSM-7000F (Jeol Ltd., Tokyo, Japan) and Tescan Vega-3 LMU (TESCAN Brno, s.r.o., Czech Republic) for selected heat treated material states in order to correlate the obtained mechanical properties with material characteristics. Quantitative characterization of secondary phase precipitates (i.e., mean particle diameter and precipitate area fraction) was performed using software ImageJ (version 1.46, National Institutes of Health, Bethesda, MD, USA). Because of the irregular shape of precipitates, the particle diameter was calculated as the Feret mean diameter [29].

## 3. Results and Discussion

### 3.1. Microstructure Evolution during Thermal Ageing

**Figure 3:**
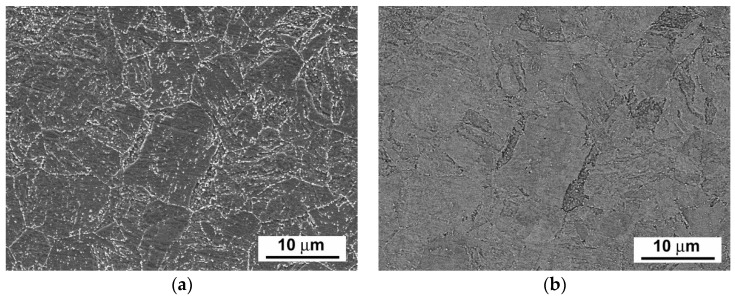
SEM images of investigated P92 steel in N&T material condition visualized by (**a**) secondary electrons and (**b**) back-scattered electrons.

**Figure 4:**
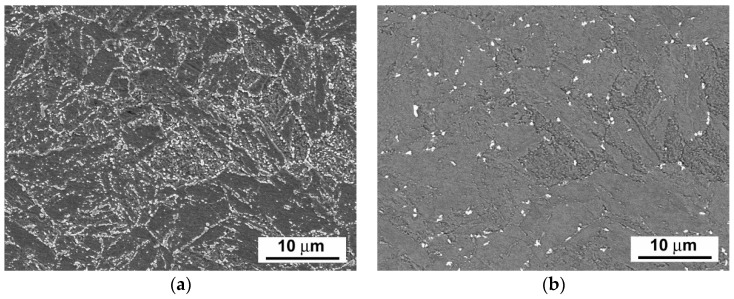
SEM images of investigated P92 steel in thermally aged material condition (600 °C/2.5 kh) visualized by (**a**) secondary electrons and (**b**) back-scattered electrons.

**Figure 5:**
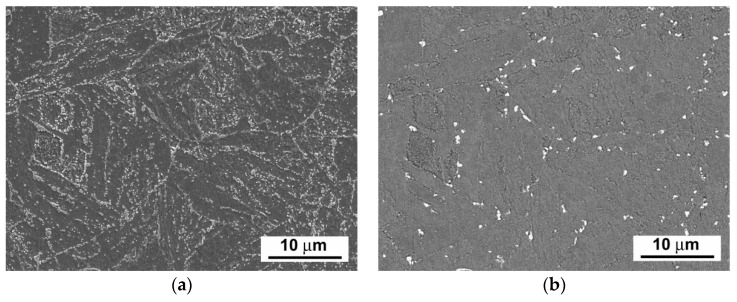
SEM images of investigated P92 steel in thermally aged material condition (600 °C/5 kh) visualized by (**a**) secondary electrons and (**b**) back-scattered electrons.

Figure 3, Figure 4 and Figure 5 show scanning electron microscope (SEM) images of P92 steel in various material conditions, i.e., N&T, 600 °C/2.5 kh, and 600 °C/5 kh, respectively.

Figure 3a, Figure 4a and Figure 5a represent SEM microstructural visualizations obtained using secondary electrons imaging (SEI) contrast, which is commonly used for detailed morphological analyses of material microstructures. Here, it demonstrates the effect of thermal ageing at 600 °C on P92 steel microstructure evolution. In order to differentiate between coarse Cr_23_C_6_-based carbides and Fe_2_W-based Laves phase, SEM visualizations were performed using back-scattered electrons (BSEs) contrast, which is highly sensitive on the average atomic number of a phase. This SEM/BSE technique has also been widely used by several other authors, e.g., [16,17,18,19], in order to identify reliably the Laves phase precipitates in 9 wt.% Cr steels. The corresponding SEM micrographs are shown in Figure 3b, Figure 4b, and Figure 5b. The Cr_23_C_6_-based carbides with a lower average atomic number than Fe_2_W-based Laves phase show indistinct grayish contrast, which disabled their identification on the BSE images. Thus, only the Laves phase particles with a much higher molecular mass than Cr_23_C_6_-based carbides could be identified on the BSE images thanks to their sharp bright contrast. The complementary image analyses of Cr_23_C_6_-based carbides in thermally aged material states were made on SEI images excluding the Laves phase particles identified on BSE images. The performed image analyses of Figure 3, Figure 4 and Figure 5 indicated the mean particle diameter of Cr_23_C_6_-based carbides for the initial N&T material state to be around 200 nm. However, after thermal ageing at 600 °C for 2.5 kh and 5 kh, the sizes of Cr_23_C_6_-based carbides increased to 233 nm and 242 nm, respectively. The mean particle diameters of the additionally precipitated Laves phase in thermally aged conditions were estimated to be 453 nm and 457 nm for 2.5 kh and 5 kh long thermal expositions, respectively. Thus, it can be concluded that, compared with the initial N&T material state with no Laves phase precipitates in microstructure, both thermally aged material states show remarkable coarsening of secondary phase precipitates. This observation clearly demonstrates the origin of thermal embrittlement of investigated P92 steel, and fairly agrees with the results of other published studies, e.g., [30,31,32], indicating that the most significant microstructural changes (i.e., gradual recovery of tempered martensitic lath microstructure, coarsening of Cr-rich M_23_C_6_ carbides, and precipitation and coarsening of W/Mo-rich Laves phase) accompanied by mechanical properties’ degradation (especially impact toughness) occur in 9 wt.% Cr steels during the first several thousand hours of their high-temperature exposure. However, although generally good qualitative agreement can be stated in terms of thermally-induced microstructural changes of grade 92 steels, quantitative variations of microstructural characteristics and material properties should be still considered owing to possible heat-to-heat variations including allowable variations of chemical composition and material processing conditions. Specifically, the coarsening behavior of the Laves phase can be strongly influenced by the chemical composition and other metallurgical factors of a specific heat (i.e., melt or product). For instance, Wang et al. [33] performed quantitative measurements of the Laves phase in grade 92 steel thermally aged at 650 °C, and they reported the mean Laves particle diameter of about 425 nm after the ageing for 8000 h. In contrast, Sun et al. [34] investigated the evolution of the microstructures and mechanical properties of grade 92 steel during ageing at 650 °C for up to 11,000 h and reported the variation of average diameter of the Laves phase from about 600 nm to about 1200 nm for the ageing times of 1000 h and 11,000 h, respectively. The coarsening behavior of Cr_23_C_6_-based carbides characterized in the present investigation fairly correlates with statistical predictions of Zielinski et al. [35] for grade 92 material aged at 600 °C. All these examples demonstrate that quantitative precipitation behavior of various heats of grade 92 material can vary, thus there is a need for individual characterization of each experimental material.

### 3.2. Effect of Thermal Ageing on Mechanical Properties

**Figure 6:**
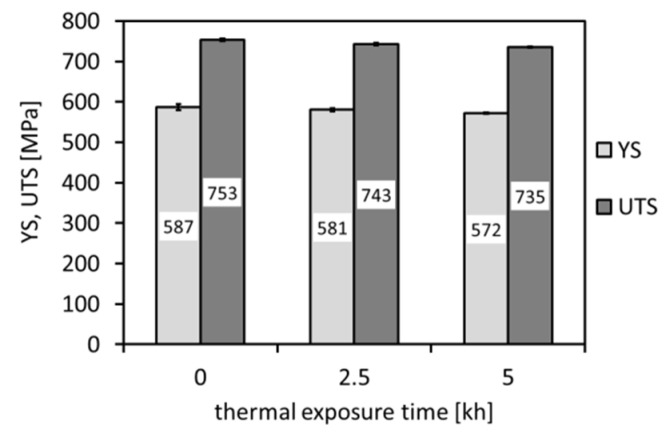
Effect of thermal exposure time at 600 °C on strength properties of investigated P92 steel.

**Figure 7:**
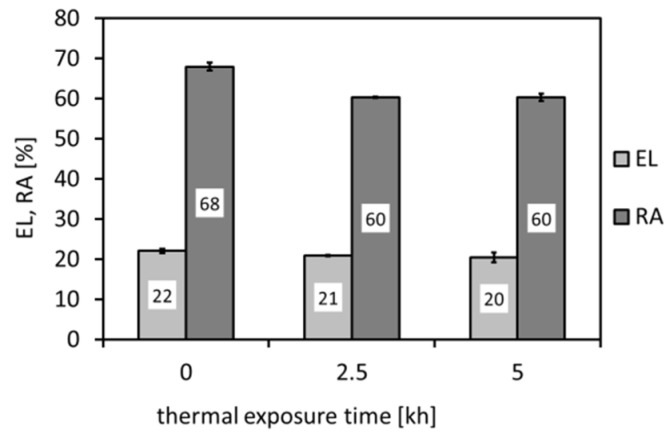
Effect of thermal exposure time at 600 °C on deformation properties of investigated P92 steel.

**Figure 8:**
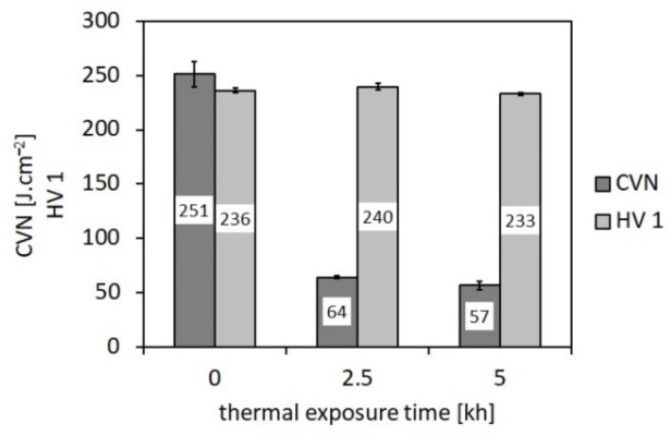
Effect of duration of thermal ageing of investigated P92 steel at 600 °C on impact toughness and hardness.

Figure 6, Figure 7 and Figure 8 show individual mechanical properties determined for the investigated P92 material in dependence of its thermal exposure time at 600 °C. In Figure 6 and Figure 7, the dependencies of strength (yield stress, ultimate tensile strength) and deformation properties (total elongation, reduction of area) are shown, respectively.

From Figure 6 and Figure 7, it can be concluded that the applied thermal exposure had only a small effect on both the strength and deformation properties obtained from quasi-static room-temperature tensile tests. Figure 8 demonstrates the effect of thermal exposure on the values of Charpy V-notch (CVN) impact toughness and Vickers hardness (HV1).

**Figure 9:**
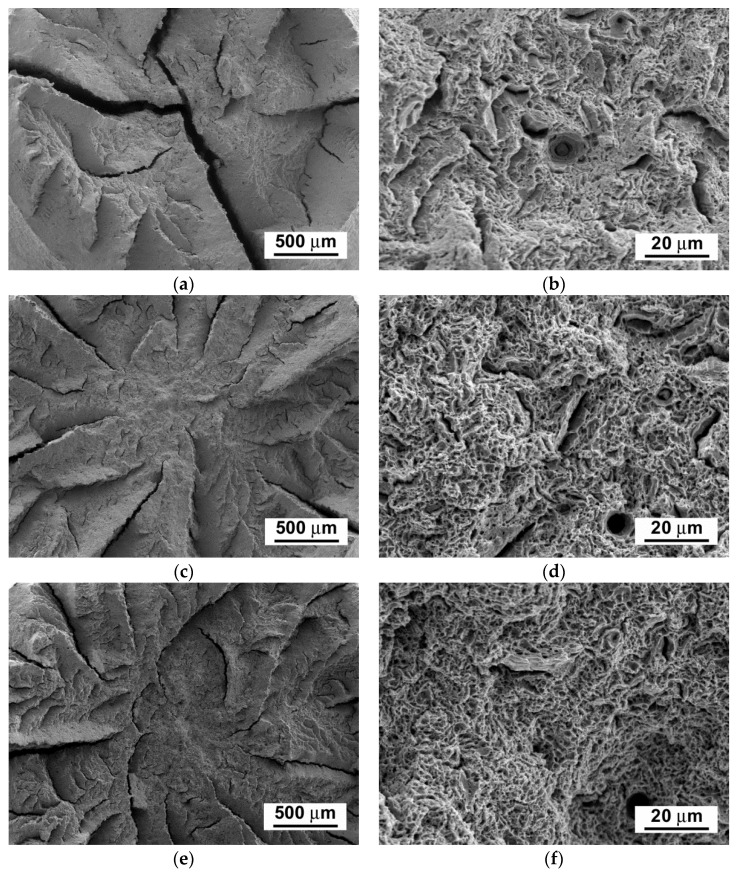
SEM fractographs of fractured tensile test specimens after quasi-static tensile tests of P92 steel in various material conditions: (**a**,**b**) N&T, (**c**,**d**) 600 °C/2.5 kh, and (**e**,**f**) 600 °C/5 kh.

**Figure 10:**
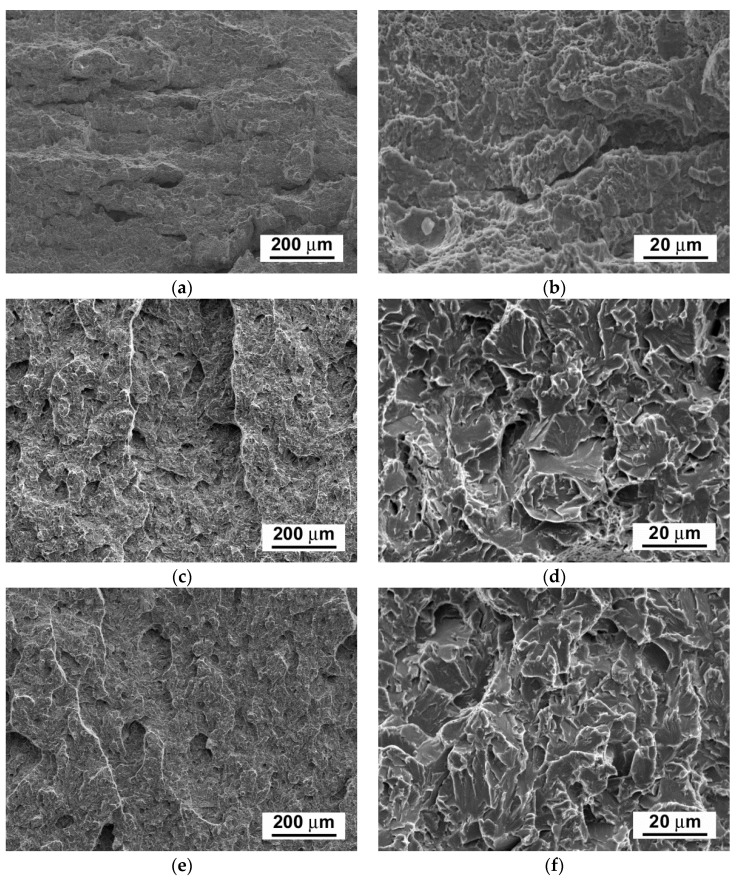
SEM fractographs of fractured impact toughness test specimens after dynamic impact bending tests of P92 steel in various material conditions: (**a**,**b**) N&T, (**c**,**d**) 600 °C/2.5 kh, and (**e**,**f**) 600 °C/5 kh.

Figure 8 shows that, with increasing ageing time, the hardness values show only negligible variations, whereas the CVN impact toughness exhibits a sharp decrease already after 2500 h of the thermal exposure. With increasing ageing time to 5000 h, the impact toughness does not exhibit further rapid decreasing. This result correlates well with the above presented microstructural observations (Figure 3, Figure 4 and Figure 5). The observed variations of room-temperature mechanical properties of our thermally aged P92 steel (Figure 6, Figure 7 and Figure 8) can be compared with other published data, e.g., in [36,37]. Zielinski et al. [36] reported similar variations of room-temperature mechanical properties for grade 92 steel aged at 600 °C. Moreover, both works [36,37] demonstrated high stability of room-temperature tensile properties of their P92 material up to approximately 30 kh of ageing time at 650 °C. Concerning the dependence of impact energy on the ageing time, a sharp drop was reported already after the ageing time of 300 h, which has been mainly attributed to early precipitation of the Laves phase [36], as also observed in the work of [18]. The obtained values of mechanical properties (Figure 6, Figure 7 and Figure 8) are correlated with corresponding characteristics of fracture surfaces of broken test specimens in Figure 9 and Figure 10.

The fracture surfaces of broken test specimens after the tensile tests show ductile dimple tearing fracture micro-mechanisms for all examined material states (Figure 9b,d,f), whereas the fracture surfaces of broken test specimens after the impact bending tests show a transition from ductile dimple tearing micro-mechanism in the initial N&T material state (Figure 10b) to transgranular quasi-cleavage in both thermally aged conditions (Figure 10d,f). Our obtained findings are in good agreement with the findings of other authors [30,31,32], who also found that the most significant changes in microstructures and mechanical properties of power-plant steels occur during the first few thousand hours of their thermal exposition and, afterwards, they undergo only very slow degradation owing to their high thermal stability at the considered temperatures. However, the observed sharp drop in impact toughness during the first time periods of thermal exposure represents a potentially serious issue for industrial application of these materials. Therefore, searching for feasible ways of impact toughness restoration is a timely topic.

### 3.3. Effect of Various Rejuvenation Heat Treatments on Mechanical Properties

**Figure 11:**
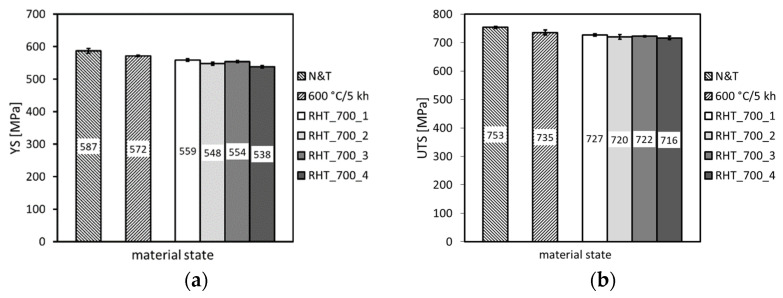
The effect of various heating up and cooling down conditions of the rejuvenation heat treatment at 700 °C for 1 h on (**a**) yield stress and (**b**) ultimate tensile strength.

**Figure 12:**
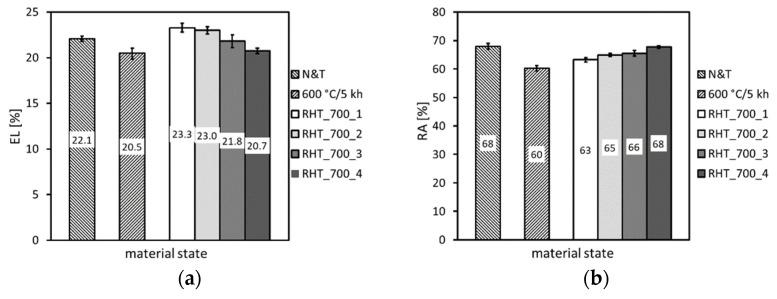
The effect of various heating up and cooling down conditions of the rejuvenation heat treatment at 700 °C for 1 h on (**a**) total elongation and (**b**) reduction of area.

**Figure 13:**
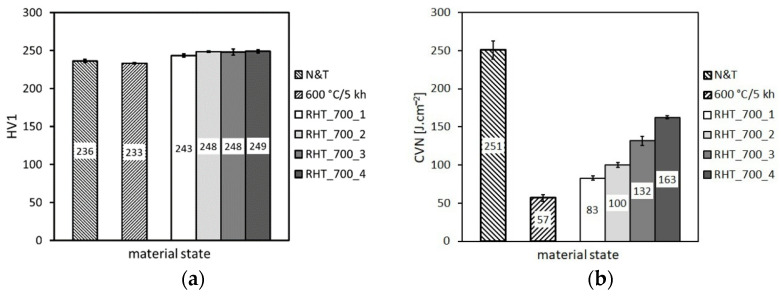
The effect of various heating up and cooling down conditions of the rejuvenation heat treatment at 700 °C for 1 h on (**a**) hardness and (**b**) impact toughness.

Zhong et al. [26] reported that the impact toughness of the already brittle P92 steel aged at 600 °C for 2035 h could be restored to the original level by reheating at 700 °C for 1 h, with the Laves phase barely changed. However, no details about the heating up and cooling down conditions were given in [26]. Therefore, in our present work, we have examined four various RHTs at 700 °C in order to examine various heating up and cooling down conditions of the rejuvenation procedure, as detailed in Table 2. The obtained mechanical properties are shown in Figure 11, Figure 12 and Figure 13.

**Figure 14:**
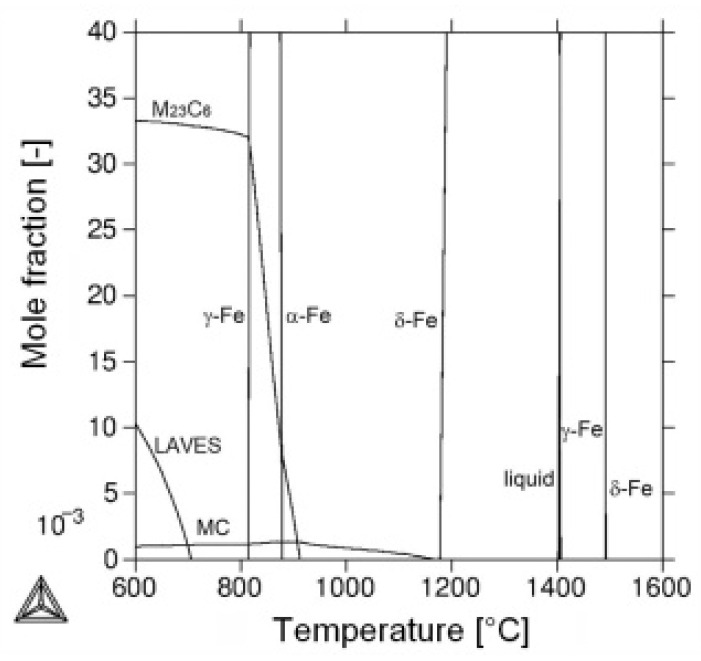
Calculated temperature dependence of mole fractions of equilibrium phases for investigated P92 steel.

From the results presented in Figure 11, Figure 12 and Figure 13, it can be concluded that the various heating up and cooling down conditions of the rejuvenation heat treatment (700 °C/1 h) of thermally aged (600 °C/5 kh) P92 steel resulted in rather small effects on strength properties (Figure 11), deformation properties (Figure 12), and hardness (Figure 13a). However, the effects of various heating up and cooling down conditions of the rejuvenation heat treatment (700 °C/1 h) of thermally aged (600 °C/5 kh) P92 steel on the resulting CVN impact toughness values were remarkable (Figure 13b). The slow heating up and slow cooling down with furnace (i.e., RHT_700_1, see Figure 2 and Table 2) resulted in the least rejuvenation effect on impact toughness, whereas the rapid heating up by directly inserting the samples into a 700 °C heated furnace and rapid cooling down in water (i.e., RHT_700_4, see Figure 2 and Table 2) resulted in the most significant rejuvenation effect. This result indicates that, for reaching the maximal rejuvenation effect, rapid cooling of the material from regarded RHT temperature is necessary in order to preserve the high temperature material state as much as possible. The cooling down of the samples on still air (i.e., RHT_700_2 and RHT_700_3, see Figure 2 and Table 2) was found to have medium rejuvenation effects on impact toughness (Figure 13b). The rapid heating up of the samples via their direct inserting into a 700 °C heated furnace (RHT_700_3) was found to be more efficient for improving the impact toughness than slowly heating up the samples with a furnace (RHT_700_2). By analyzing the results obtained using various heating up and cooling down conditions of the RHTs performed at 700 °C, it can be concluded that the rapid heating up by directly inserting the samples into a 700 °C heated furnace and rapid cooling down in water (i.e., RHT_700_4) represent the most suitable heating up and cooling down conditions of the samples rejuvenated at 700 °C for a 1 h holding time. However, despite the clear improvement in impact toughness (about 163 J·cm^−2^) after performing RHT_700_4 compared with thermally aged state (57 J·cm^−2^), the original value of the initial N&T material state (251 J·cm^−2^) could not be reached (Figure 13b). This result can be related to the fact that the used RHT temperature of 700 °C was too low to promote such microstructural changes of the P92 steel material that would lead to notable changes of its mechanical properties. This conclusion can also be supported by the results of hardness measurements (Figure 13a). Moreover, the RHT temperature of 700 °C was lower than the estimated Laves phase solvus temperature of 704 °C by thermodynamic calculation (Figure 2). The equilibrium calculation of temperature dependence of individual phase amounts in P92 steel is shown in Figure 14.

**Figure 15:**
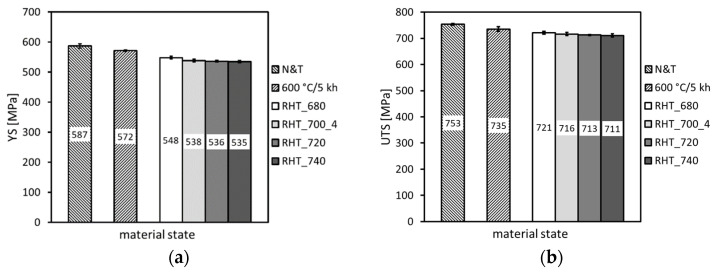
The effect of various annealing temperatures of individual rejuvenation heat treatments with most efficient heating up and cooling down conditions on (**a**) yield stress and (**b**) ultimate tensile strength.

**Figure 16:**
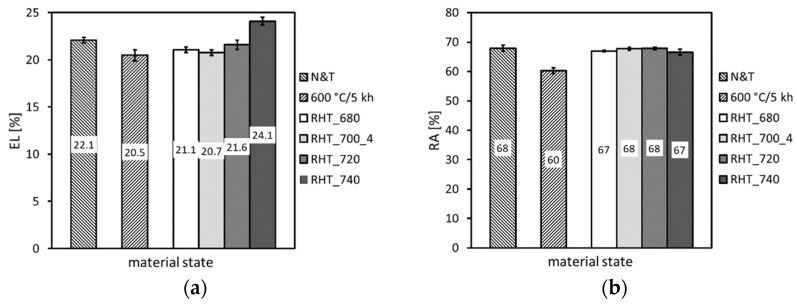
The effect of various annealing temperatures of individual rejuvenation heat treatments with most efficient heating up and cooling down conditions on (**a**) total elongation and (**b**) reduction of area.

**Figure 17:**
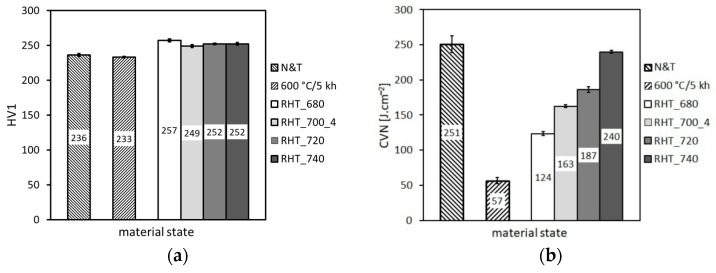
The effect of various annealing temperatures of individual rejuvenation heat treatments with most efficient heating up and cooling down conditions on (**a**) hardness and (**b**) impact toughness.

This calculation indicates that some decrease in the amount of Laves phase precipitates is to be expected by performing the RHTs at 700 °C of thermally exposed P92 material at 600 °C. However, owing to the slow diffusion kinetics of such heavy elements like tungsten and molybdenum and too short holding time at the RHT annealing temperature, these thermodynamically expected changes of the Fe_2_(W,Mo)-based Laves phase precipitation state might be practically negligible. In order to justify the obtained findings and for the sake of the complexity of present research aimed at reaching the maximal rejuvenation effect, a further three RHTs at 680 °C, 720 °C, and 740 °C with the most efficient heating up and cooling down conditions were individually performed. The effects of these RHTs on resulting mechanical properties are graphically demonstrated in Figure 15, Figure 16 and Figure 17.

**Figure 18:**
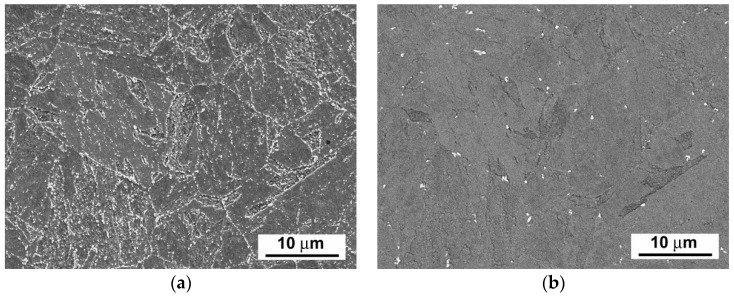
SEM microstructures of investigated P92 steel in thermally aged (600 °C/5 kh) and rejuvenated (RHT_740) material condition, visualized by (**a**) secondary electrons and (**b**) back-scattered electrons.

**Figure 19:**
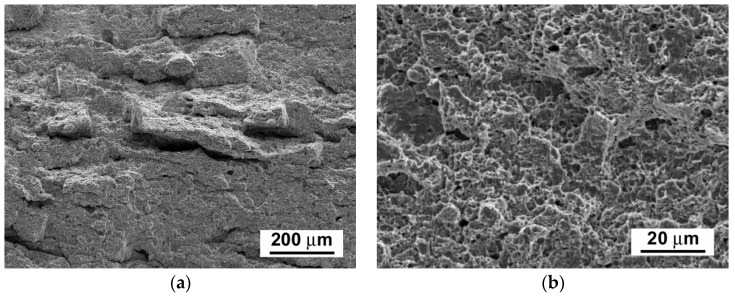
SEM fractographs of fractured impact toughness test specimen of P92 steel material after the Charpy impact bending test: (**a**) overall view on fracture surface and (**b**) detailed view on ductile dimple fracture micro-mechanism.

From the results presented in Figure 15, Figure 16 and Figure 17, it can be concluded that the various annealing temperatures (in the range from 680 °C to 740 °C) of the RHTs of thermally aged (600 °C/5 kh) P92 steel with the most efficient heating up and cooling down conditions had only marginal effects on the resulting strength properties (Figure 15), deformation properties (Figure 16), and hardness (Figure 17a). However, the effects of various annealing temperatures of the RHTs of thermally aged P92 steel on the resulting CVN impact toughness values were significant (Figure 17b). It is concluded that the maximal rejuvenation effect, i.e., restoration of impact toughness of thermally aged P92 material up to nearly original value, was reached after performing the RHT at 740 °C. The obtained improvement in impact toughness for this rejuvenated material state is correlated with its corresponding microstructural and fracture characteristics in Figure 18 and Figure 19, respectively. As all the RHTs were performed in subcritical temperature range (i.e., below the Ac_1_ temperature of P92 steel) and because of their short time duration (1 h), the effects of nano- or micro-damaging and grain size effects are considered to be insignificant.

By comparison of microstructure of P92 steel after thermal ageing at 600 °C for 5000 h (Figure 5) and that after subsequent rejuvenation at 740 °C for 1 h (Figure 18), it can be concluded that a noticeable decrease in the amount of Laves phase precipitates (by approximately 60% of their original area fraction) occurred after the rejuvenation, which is likely the most crucial effect responsible for the observed impact toughness restoration. This conclusion can be supported by the findings of Komazaki et al. [23], who found that the area fraction of the Laves phase precipitates of a certain critical size has a major effect on the impact toughness of P92 steel. The mean particle diameters of the Laves phase and Cr_23_C_6_-based carbide after the rejuvenation decreased to 395 nm and 214 nm, respectively. This result indicates the accompanying significance of particle size effects on the rejuvenation. Compared with the completely embrittled fracture surfaces corresponding to thermally aged material state (Figure 10e,f), the rejuvenation effect achieved by the most efficient annealing conditions (740 °C/1 h) can also be clearly demonstrated on the fracture surfaces, showing full restoration of the ductile dimple tearing fracture micro-mechanism (Figure 19). The importance of applying rapid cooling down from the rejuvenation temperature to preserve secondary phase dissolution correlates well with the findings of Sello and Stumpf [24], who investigated the effects of Fe_2_Nb type Laves phase on AISI 441 ferritic stainless steel. Based on the findings of the present investigation, it can be highlighted that partial dissolution of the Fe_2_W-based Laves phase precipitates in thermally aged P92 steel is sufficient for obtaining mechanical properties closely approaching the original values of N&T material.

## 4. Summary and Conclusions

In this work, the effects of various conditions of short-term rejuvenation heat treatments of long-term thermally aged P92 boiler steel on its room-temperature mechanical properties’ restoration were investigated. Here are the main conclusions.

- Long-term ageing of investigated P92 steel at 600 °C up to 5000 h led only to small changes in its room-temperature tensile properties and hardness, whereas its Charpy impact toughness was significantly deteriorated. The sharp drop in impact toughness occurred already after 2500 h ageing at 600 °C and, afterwards, i.e., following the increase in the ageing time, it did not experience any further notable decrease. The main reason for the impact toughness deterioration was ascribed to thermal embrittlement caused by coarsening of secondary phase precipitates, especially the Fe_2_W-based Laves phase.
- By investigating the effects of various heating up and cooling down conditions of the rejuvenation heat treatments at 700 °C for 1 h, it was found that the rapid heating up by directly inserting examined specimens into the heated furnace and quick cooling down of the specimens in water represent the most efficient conditions for the maximization of the rejuvenation effect for the restoration of impact toughness. This result indicates that, for obtaining the maximal rejuvenation effect at the considered rejuvenation annealing temperature, rapid cooling of the material from the annealing temperature is needed in order to preserve the high temperature material state, and thus avoid reversible diffusion-controlled microstructural changes that might reduce the rejuvenation effect by slower cooling.
- By investigating the effects of various rejuvenation annealing temperatures in the range from 680 °C to 740 °C, it was revealed that the maximal rejuvenation effect for CVN impact toughness restoration of long-term thermally aged P92 steel (i.e., obtaining the CVN values closely approaching the original values corresponding to normalized and tempered material condition) was obtained at 740 °C using the most efficient, i.e., rapid heating up and fast cooling down, conditions. From the performed microstructural observations, it can be concluded that the rejuvenation effect can be ascribed to partial dissolution of the Laves phase precipitates, i.e., around 60% of their original area fraction. The rejuvenation effect was also correlated with fractographic observations, indicating full restoration of the ductile dimple fracture micro-mechanism.

## Figures and Tables

**Table 1:** Chemical composition (wt.%) of the investigated material.

Material	C	Si	Mn	Cr	Mo	W	Al	Ni	Cu	V	Nb	Co	Fe
P92	0.13	0.37	0.42	9.39	0.28	1.54	0.006	0.29	0.09	0.23	0.07	0.01	balance

**Table 2:** Rejuvenation heat treatment (RHT) conditions.

RHT Denotation	Annealing Temperature/Time	Heating Up (Approx. Rate)	Cooling Down (Approx. Rate)
RHT_700_1	700 °C/1 h	with furnace (4.7 °C/min)	with furnace (0.7 °C/min)
RHT_700_2	700 °C/1 h	with furnace (4.7 °C/min)	on still air (100 °C/min)
RHT_700_3	700 °C/1 h	inserting at 700 °C (350 °C/min)	on still air (100 °C/min)
RHT_700_4	700 °C/1 h	inserting at 700 °C (350 °C/min)	into water (700 °C/min)
RHT_680	680 °C/1 h	inserting at 680 °C (350 °C/min)	into water (700 °C/min)
RHT_720	720 °C/1 h	inserting at 720 °C (350 °C/min)	into water (700 °C/min)
RHT_740	740 °C/1 h	inserting at 740 °C (350 °C/min)	into water (700 °C/min)

## Data Availability

Not applicable.
